# Vehicular Emission Inventory and Reduction Scenario Analysis in the Yangtze River Delta, China

**DOI:** 10.3390/ijerph16234790

**Published:** 2019-11-29

**Authors:** Xiaowei Song, Yongpei Hao

**Affiliations:** 1 College of Resources and Environment, Shanxi University of Finance & Economics, Taiyuan 030006, China; songxiaowei@sxufe.edu.cn; 2 School of the Environment, Nanjing University, Nanjing 210046, China; 3 Ministry of Education Key Laboratory for Coastal and Island Development, School of Geographic & Oceanographic Sciences, Nanjing University, Nanjing 210046, China

**Keywords:** vehicular pollution, emission inventory, scenario analysis, life-cycle analysis, YRD

## Abstract

Vehicular emissions have become an important source of air pollution, and their effective reduction control is essential to protect the environment. The aim of this study was to establish multi-year vehicular emission inventories for ten important air pollutants and to analyze emission control policy scenarios based on these inventories. The inter-annual emission analysis results showed that the ten pollutant emissions had different change trends during the past decade. The emissions of CO, non-methane volatile organic compounds (NMVOC_S_), NO_x_, PM_2.5_, PM_10_, and CH_4_ tended to increase first and then decrease, but the years in which they began to decrease varied; the emissions of CO_2_ and NH_3_ showed the most significant growth trends, increasing by 567% and 4004% in 2015 compared with 1999, while the emissions of N_2_O and SO_2_ showed a general increasing trend and decreased obviously in a certain year. Eight scenarios based on emission inventories were designed; compared with the BAU scenario, the ESV scenario was the most effective policy to control NO_x_, PM_2.5_, and CH_4_ emissions; the radical AER scenario could decrease the vehicular emissions of CO, NMVOCs, PM_10_, CO_2_, N_2_O, and NH_3_; and the RFS scenario could reduce vehicular SO_2_ emissions significantly by 93.64%.

## 1. Introduction

The rapid development of the global economy has caused the number of vehicles in many countries, particularly developing countries, to grow rapidly. Vehicular pollution is one of the main sources of air pollution with serious effects on climate change [1,2] and human health [3,4]. Zheng et al. (2015) [5] pointed out that transportation has become an important sector for the reduction of greenhouse gas (GHG) emissions, because the continuous growth in the number of vehicles has resulted in substantial amounts of burned fuel and emissions of massive amounts of GHGs in China. Lang et al. (2018) [2] noted that particulate matter (PM) emitted by mobile sources could directly increase atmospheric particulate matter with a diameter less than 2.5 μm (PM_2.5_). Sun et al. (2019) [6] reported that the rapid increase in the number of vehicles is a major factor that has affected the urban ambient air quality in China in recent years. In summary, vehicle-related pollutants have gradually become an important concern for air pollution control [7]. Additionally, with the constant expansion of cities, air pollutants emitted in cities has affected the whole region including neighboring cities. Air pollution has gradually changed from a local (city) problem to a regional one. Thus, there is an urgent need to study vehicular emissions and emission reduction strategies from a regional angle.

Studies on vehicular emissions and control strategies have been aimed at identifying the dominant vehicular-related pollutant sources and their spatial and temporal distributions to allow for the establishment of emissions control and environmental management tailored to the current situation. Vehicular emission inventory is established by combining the vehicle population, vehicle activity, and emission factors. Vehicular pollutant emission factor models applicable to different situations and ranges have been established using different simulation methods in Europe, the USA, and other countries [8,9,10,11,12,13]. These models provide good estimates of pollutant emission factors and allow emission inventories to be established. In China, some researchers have studied the emission inventory and emission characteristics of vehicular pollution in regions with a developed economy along the east coast of China, such as the Beijing–Tianjin–Hebei Region [14] and the Pearl River Delta [15]. Based on the establishment of a vehicular emission inventory, research on vehicular emission reduction measures are essential and useful. Some scholars have also evaluated the effects of vehicular emission reduction based on the scenario analysis method. Liu et al. (2017) [15] predicted the emissions of air pollutants and GHGs by 2020 based on the no-control scenario and then calculated emission reductions based on the design of five vehicular emission reduction scenarios in the Pearl River Delta, concluding that updated emission standard scenarios were best to reduce air pollutants and GHGs substantially. Guo et al. (2016) [16] used the scenario analysis method to predict the reduction potentials of CO, NO_x_, HC, and PM_10_ under five control strategies and policies in the Beijing–Tianjin–Hebei region during 2011–2020 and concluded that the scenario of eliminating high-emission vehicles can reduce emissions more effectively in the short term than in the long term, especially in Beijing, and that the integrated scenario considering all of the control measures would achieve the maximum reduction potential of emissions.

**Figure 1:**
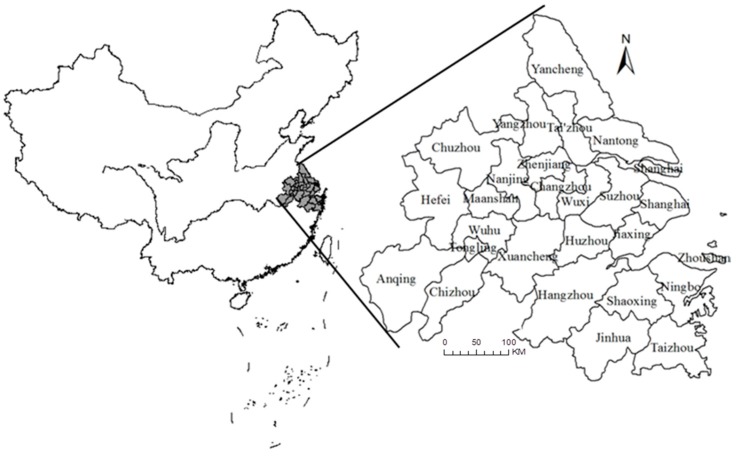
Location of the Yangtze River Delta with the administrative divisions shown.

As one of the fastest developing regions in China, the Yangtze River Delta (YRD) is the most dynamic and open economy and the strongest innovation area of any Chinese region. The YRD, geographically comprising the Shanghai municipality, nine prefecture-level cities in Jiangsu Province, eight prefecture-level cities in Zhejiang Province, and eight prefecture-level cities in Anhui Province (Figure 1), constitutes only 2.2% of China’s total area and contains 11% of the resident population and contributed to 19.8% of the total national gross domestic product in 2015 [17]. The number of vehicles in the YRD has increased rapidly in recent years (this region comprised 15% of China’s total vehicle population in 2015), and vehicular pollution has become a serious problem. There is an urgent need to study vehicular pollutant emissions and emission reduction strategies to maintain social development and urbanization in the YRD. Deng (2011) [18] established a vehicular emission inventory for the YRD (Shanghai, Jiangsu, and Zhejiang) for 2006–2008 using the Computer Programme to Calculate Emissions from Road Transport (COPERT IV) model and assessed the emission characteristics. Song et al. (2016) [19] calculated the vehicular pollutant (CO, non-methane volatile organic compounds (NMVOCs), NO_x_, black wood charcoal (BC), organic carbon (OC), PM_2.5_, and PM_10_) emissions in the Pan-YRD using the COPERT IV model from 1999 to 2013 and analyzed them from the aspects of time variation trend, spatial distribution characteristics, and pollutant emission characteristics of different vehicle types. Several studies have focused on vehicular emission reduction strategies for the YRD. Wang (2018) [20] established the emission inventory of motor vehicles in the YRD in 2015 and comprehensively analyzed the characteristics of vehicle pollution emissions and then assessed the emission reduction effects of CO, NO_x_, HC, and PM of the five policies in 2015, concluding that the elimination of yellow-label vehicles (i.e., gasoline vehicles with emission levels lower than the State I emission standards and diesel vehicles with emission levels lower than the State III emission standards) and old cars (vehicles that do not meet the State IV emission standards) had the best effect on reducing emissions.

Previous studies have presented some detailed information on vehicular emissions and provided the basis for vehicular pollutant reduction in the YRD. However, more studies are needed because many new vehicle control measures have been implemented in this area. Thus, the calculation of vehicular emissions and the assessment of control policy effects must include the implications of these new control measures. Additionally, these previous evaluations were not sufficiently comprehensive because life-cycle analyses of vehicles using new sources of energy were not performed. Therefore, it is critical to investigate in a comprehensive and integrated manner vehicular pollutant emissions and emission reduction strategies in the YRD.

The purposes of this study were to investigate the time variation trends (from 1999 to 2015) of vehicular emissions and to assess the emission reduction effects of different control measures in the YRD. Ten types of vehicular pollutants were included: CO, NMVOC, NO_x_, PM_2.5_, PM_10_, CO_2_, CH_4_, N_2_O, NH_3_, and SO_2_. Vehicular emission trends were analyzed to ensure the integrity of the regional pollutant emission inventory. The vehicular pollutant emission analysis results were used to define eight pollutant emission reduction scenarios (business as usual (BAU), high standard replacement (HSR), raising fuel standards (RFS), elimination of substandard vehicles (ESV), public transport priority (PTP), alternative energy replacement (AER), elimination of motorcycles (EMC), and integrated scenario (IS)), and the effects of implementing these scenarios were evaluated. A radical alternative energy replacement (RAER) scenario was evaluated using life-cycle evaluation. Finally, the Monte Carlo method was used to perform uncertainty analysis of the emission inventories. The results are also expected to be relevant to vehicular emission reduction strategies in other cities and regions.

## 2. Methodology

### 2.1. Emission Estimates

Vehicular emissions were calculated based on vehicle populations, annual mean vehicle kilometers travelled (VKT), and emission factors using the following:

(1) $$Q_{m,n\;}=\;\underset{i}{\sum }\underset{j}{\sum }\left(P_{m,i,j}\times VKT_{m,i}\times EF_{i,j,n}\right)$$

where *Q_m,n_* is the amount of pollutant *n* emitted in area *m* each year, *P_m,i,j_* is the number of vehicles in category *i* with emission standard *j* in area *m*, *VKT_m,i_* is the mean annual VKT (km) for vehicles in category *i* in area *m*, and *EF_i,j_,_n_* is the emission factor (g/km) for pollutant *n* emitted by vehicles in category *i* with emission standard *j*. The vehicle categories in this work included passenger car (PC), bus (BUS), light-duty vehicle (LDV), heavy-duty truck (HDT), and motorcycle (MC).

#### 2.1.1. Vehicle Population

The vehicle populations for the different vehicle types in each city in the YRD between 1999 and 2015 were obtained from local statistical yearbooks [17,21,22,23,24] and statistical bulletins for national economic and social development for each city. The statistical yearbook of each province did not contain vehicle populations of prefecture-level cities prior to 2002. The PC data before 2002 for each city were estimated using the Gompertz model [16,25]. A Gompertz curve was drawn using the PC population and per capita gross domestic product (GDP) for each city between 2002 and 2015. Next, the PC populations for the cities between 1999 and 2001 were estimated from the per capita GDP for the cities between 1999 and 2001. The BUS, LDV, HDT, and MC vehicle numbers between 1999 and 2001 were estimated from the regressions of vehicle numbers between 2002 and 2015.

New vehicles must follow any new standard once it has been implemented [26,27]. The dates of introduction of the vehicular emission standards of each city are summarized in Table S1. The vehicle populations when the emission standards for the different vehicle types were implemented were calculated using the annual number of new vehicle registrations, vehicle survival rates, and dates of implementation of the vehicular emission standards in the different cities using the method of Lang et al. (2014) [28] and Sun et al. (2016) [27] as expressed in Equation (2):

(2) $$P_{m,i,j}\;=\;\{\begin{array}{c} \underset{n}{\sum }N_{m,i,j}\cdot r_{y-k}\;j\;\neq \;S0\\ P_{m,i,total}-\;\overset{S5}{\underset{S1}{\sum }}\underset{n}{\sum }N_{m,i,j}\cdot r_{y-k}\;j\;=\;S0 \end{array}$$

where *N_m,i,j_* represents the newly registered population of vehicle *i* meeting emission standard *j* in area *m* (S0 to S5 are emission standards for State 0 to State V, respectively), *P_m,i,total_* is the total vehicle population *i* in area *m*, *k* is the year for newly registered motor vehicles, *y* is the year of interest (study year), *n* is the year of implementation of the emission standard of interest, and *r* is the vehicle survival rate. Vehicle survival rates were obtained from References [27,29,30] and are summarized in Table S2.

**Figure 2:**
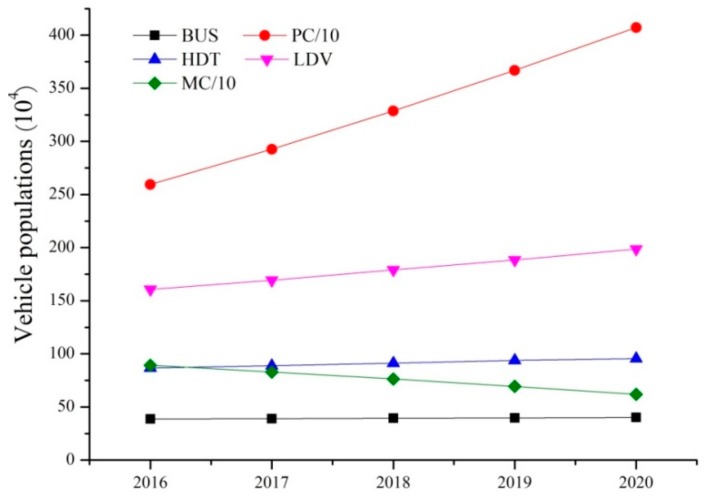
Predicted values of the vehicle population in the study region between 2016 and 2020.

The categorical prediction method was used to predict the vehicle population of every city in the study area between 2016 and 2020. The PC population was affected by many factors, particularly per capita income. Numerous studies [31,32] have demonstrated that the PC population conforms to the Gompertz model curve, i.e., the vehicle retention rate increases with the per capita income and follows an S-shaped curve. The vehicle retention rate increases to a maximum and then decreases until it reaches a plateau. The PC population was predicted using the Gompertz function as follows:

(3) $$V\left(x\right)\;=\;\gamma e^{\alpha^{e^{\left(\beta x\right)}}}$$

where *V*(*x*) represents the PC population (units/thousand people), *x* is the per capita disposable income (yuan/person), *γ* is the PC retention rate plateau (values obtained from References [16,33], and a value of 0.6 was selected), and *α* and *β* are parameters indicating the model trend, obtained by fitting a line to a plot of the PC retention rate data (the PC population divided by the demographic data) for 1999–2015 against per capita disposable income. The per capita disposable incomes for 26 cities (for 2016–2020) were projected from equivalent incomes for 1999–2015. The PC population retention rates for each city for 2016–2020 were predicted using Equation (3). The populations of the cities in 2016–2020 were predicted using the grey model. The PC populations of the cities in 2016–2020 were calculated from these data. The HDT, LDV, BUS, and MC populations were predicted using the regression curve method. The YRD vehicle populations in 2016–2020 (shown in Figure 2) were then calculated. The same method was used to predict the population of newly registered vehicles in each city for 2016–2020. The future age distributions for each vehicle type were calculated from the vehicle survival rates (Table S2), assuming that each vehicle met the relevant emission standard in the registration year. Thus, the vehicle populations implementing different emission standards in each city from 2016 to 2020 were determined.

#### 2.1.2. Vehicle Kilometers Travelled (VKT)

The mean annual VKT, a major vehicle activity level indicator, can influence vehicular emissions. The VKT data for different vehicle types were not available because VKT data are not included in official statistical records. The VKT values for Shanghai, Nanjing, Hangzhou, and Hefei were obtained from previous studies [18,25,28,34,35,36,37,38], and missing data were obtained from linear interpolations [14]. The missing data in other cities were obtained by making reasonable assumptions referring to the cities with a similar level of economic development and vehicle population given that both the VKT values and vehicle population are strongly related to economic activity [25,39]. The partial mean annual VKT data of each vehicle type are shown in Table S3.

The mean annual VKT data of different vehicle types in each city in the YRD between 2016 and 2020 were predicted. It was previously found that the average annual VKT for PC correlates with its retention rate [39], i.e., the annual VKT decreases as the retention rate increases each year. The PC retention rate in a city is expected to continue to grow. A (index) correlation between the annual PC VKT and retention rate for each city was therefore established using the annual VKT of PC for each city in the YRD from 1999 to 2015. The annual VKT of PC from 2016 to 2020 was determined from the PC retention rates for 2016–2020 as predicted using the Gompertz curve. The annual VKT values of HDT, BUS, and LDV are related to commercial activities and increase with economic growth. Therefore, the annual VKT values of HDT, LDV, and BUS for 2016–2020 were predicted using the elasticity coefficient method. The elastic coefficients for the annual average growth rate of the VKT of HDT, LDV, and BUS and the annual GDP growth rates for 1999–2015 were calculated. The annual average growth rate of GDP for each city for 2016–2020 was predicted from the relevant predictions of the potential GDP growth rates made by the Chinese Academy of Social Sciences. The annual VKT values of HDT, LDV, and BUS for 2016–2020 were then calculated. The annual VKT of MC will decrease in the next few years, because motorcycle use limits are being applied in each city. The predicted annual VKT data of different vehicle types are shown in Table S4.

#### 2.1.3. Emission Factors

The COPERT IV (v11.2) model was used to calculate the vehicular emission factors. This model needs many input parameters related to vehicular emissions such as the vehicle type, fuel quality, mean driving speed, and meteorological data. The vehicle types (i.e., PC, LDV, HDT, BUS, and MC) used in the COPERT IV model are different from those used in China. The method used to convert among the two types is presented in Table S5. The mean driving speeds for the different vehicle types were obtained from previous publications [14,18,26,35,40,41,42]. Fuel quality parameters were obtained from Chinese national and local fuel standards. The gasoline and diesel sulfur contents are shown in Table S6. The meteorological data (including the maximum and minimum air temperature and humidity) were from the Chinese Meteorological Yearbook [43].

The emission factors for the different vehicle types in different cities were predicted using the COPERT IV model using predicted values for the parameters as required by the model [44]. The predicted parameters included fuel consumption, temperature, humidity, etc. Gasoline and diesel consumption were predicted using the linear regression method. There is no ideal method to predict temperature and humidity for short periods on a monthly basis. The predicted maximum temperature, minimum temperature, and mean humidity for each month between 2016 and 2020 were defined as the mean maximum temperature, minimum temperature, and mean humidity, respectively, in the same months between 1999 and 2015. These predictions and the results of previous studies [45,46,47,48] allowed vehicle pollutant emission factors for 2016–2020 to be estimated.

### 2.2. Design of Reduction Scenarios

Vehicular pollution reduction measures are commonly used to achieve a reduction of air pollutants in the YRD such as the elimination of yellow-label vehicles, updated emission standards, and improving fuel quality. In this part, eight reduction scenarios were designed, and the details of these scenarios are described below:

Business as usual (BAU) scenario: The BAU scenario involved the use of existing vehicular emissions control measures, the natural elimination of vehicles, and non-implementation of any additional emission reduction measures.

High standard replacement (HSR) scenario: The HSR scenario was defined in terms of the application of higher emission standards to new vehicles to meet vehicular pollutant emission standards set by the Chinese Ministry of Environmental Production in the YRD in the future (see Table S7). Other control measures were the same as those of the BAU scenario.

Raising fuel standards (RFS) scenario: Implementing new oil quality standards could cause an immediate decrease in vehicular emissions. Decreasing the sulfur content of vehicle fuel is essential to decrease vehicular emissions of sulfur. The sulfur contents of oil products were predicted from the plans made by each city to implement vehicle oil standards. In the RFS scenario, it was assumed that gasoline vehicles met the State V vehicular fuel standard in 2015 and that diesel vehicles met the State VI vehicular fuel standard in 2018 in Shanghai and the cities of Jiangsu and Zhejiang. It was assumed that gasoline vehicles met the State V vehicular fuel standard in 2017 and that diesel vehicles met the State VI vehicular fuel standard in 2019 in cities in Anhui. The vehicle population and annual VKT were the same as the BAU scenario. Emission factors were obtained using the COPERT IV model by entering the corresponding fuel standard parameters.

Elimination of substandard vehicles (ESV) scenario: Elimination of yellow-label vehicles and heavily polluting vehicles will effectively decrease vehicular pollutant emissions. We assumed that all yellow-label vehicles and pre-State I MC were eliminated by 2015, that gasoline State I vehicles and State II vehicles and diesel State III vehicles were eliminated by 2018, and that gasoline State III vehicles and State I and State II MC will be eliminated by 2020 in Shanghai. We assumed that yellow-label vehicles and pre-State I MC were eliminated by 2017 and that gasoline State I and State II vehicles, diesel State III vehicles, and State I and State II MC will be eliminated by 2020 in cities in Jiangsu, Zhejiang, and Anhui. The emission factors and annual VKT values were the same as the BAU scenario. The total number of vehicles was maintained by assuming all the eliminated vehicles were replaced with vehicles meeting up-to-date emission standards in the year the vehicles were replaced.

Public transport priority (PTP) scenario: The development of public transport is an effective means of increasing the proportion of residents using public transport and decreasing the number of PC and MC used thus decreasing the mean PC and MC VKT. It was previously found that the annual PC VKT decreased by 1% per year as the proportion of public transportation use increased [49]. We assumed that limiting MC use would decrease the mean MC VKT by 2% per year. The PTP scenario implies that the PC and MC VKT in 2020 will be 5% and 10% lower than the BAU scenario for the YRD, respectively.

Alternative energy replacement (AER) scenario: The promotion of vehicles using new energy sources is an effective means to decrease energy consumption and pollutant emissions. However, a large proportion of the electricity in China is produced by coal-powered plants. Reductions in pollutant emissions achieved through the promotion of electric vehicles are substantially reduced if the upstream pollutant emissions are considered. Life-cycle assessment methods must be used to assess electric vehicles. Therefore, we used a conservative alternative energy replacement (CAER) scenario and a radical alternative energy replacement (RAER) scenario to assess emission reductions achieved using electric and hybrid vehicles and vehicles powered by natural gas. The CAER scenario involved 80% of BUS using alternative fuels and advanced vehicle power technologies by 2020. We assumed that electric, hybrid, and natural gas-powered BUS would account for 50%, 30%, and 20%, respectively, of all BUS in 2020, 20% of PC would be electric, and 10% would be hybrid. In the RAER scenario, we assumed that the electricity provided to the vehicles would be produced using “clean” energy. The other RAER parameters were the same as those for the CAER scenario.

Emission factors for hybrid and natural gas-vehicles were taken from existing research [45,46,47,48,50,51,52] (Table S8). The annual hybrid and natural gas-powered vehicle VKT values were the same as those for the BAU scenario. These data allowed for the estimation of emissions from hybrid and natural gas-powered vehicles in the YRD.

The life-cycle of an electric vehicle was analyzed using the Greenhouse Gases, Regulated Emissions, and Energy Use in Transportation (GREET) model developed by the National Argonne Laboratory and used in previous studies [53,54,55]. The electric vehicle life-cycle includes the fuel and material life-cycles. The fuel life-cycle has two phases, the well-to-tank (WTT) phase and the tank-to-wheels (TTW) phase, which cover raw material production, fuel supply, and driving. Energy consumption, greenhouse gas emissions, and pollutant emissions during the fuel life-cycle account for 70–90% of the total life-cycle [56]. The material life-cycle accounts for a relatively small proportion of the total life-cycle. It was difficult to obtain energy consumption and pollutant emission data for the material life-cycle because numerous materials (electric vehicle production materials) are processed and used when manufacturing vehicles. Therefore, the study focused on the electric vehicle fuel life-cycle.

#### 2.2.1. Calculating Energy Consumption

Coal-fired power plants are the most important sources of electricity in China. Therefore, the fuel life-cycle analysis was focused on pollutants emitted by coal-fired power plants. The Chinese power industry has developed rapidly in recent years, and the total power generated has increased each year. The total power generated by the Chinese power industry in 2020 is predicted to be 7.4 trillion kWh [57]. The proportions of power produced by different methods were determined from the national average power composition data. Coal-fired power plants are predicted to contribute 78% of the electricity produced in China in 2020 [58,59], assuming this is for the YRD. Energy consumption and pollutant emissions from power plants are closely related to the power generation efficiency. The International Energy Agency [58] predicted that Chinese coal-fired power plants will have a generation efficiency of 38% in 2020. Assuming this to be the same for the YRD as for China as a whole, the power generation efficiency was calculated using the following equation:

(4) $$\alpha \;=\;\frac{\beta \;\times \;\mu }{\gamma \;\times \;\pi }$$

where *α* is the coal-fired power generation efficiency (%), *γ* is the amount of standard coal used in coal-fired power plants (kg), *π* is the low calorific value of standard coal (J/kg), *β* is the amount of electricity produced by coal-fired power plant (kWh), and *μ* is the electro-thermal conversion coefficient (J/kWh).

#### 2.2.2. Calculating Pollutant Emissions

The CO_2_ emissions were estimated using the carbon balance method and were determined by adding direct CO_2_ emissions and indirect CO_2_ emissions together. The carbon in direct CO_2_ emissions were defined as the amount of carbon in the combustion products (VOCs, CO, and CH_4_) subtracted from the amount of carbon in the raw fuel. Indirect CO_2_ emissions were calculated from the VOC_s_ and CO emissions [57].

Non-combustion emissions and dust pollution were not considered because the study was focused on the combustion emissions of CO, NMVOCs, NO_x_, PM_2.5_, PM_10_, CH_4_, N_2_O, and SO_2_. The NH_3_ emissions in the upstream stage of the vehicles were excluded, because it was very difficult to estimate an NH_3_ emission factor. The SO_2_ emissions were estimated using the sulfur balance method [53]. Emissions of the other pollutants were calculated using the following equation:

(5) $$N_{WTT_{I}}=\;\underset{J}{\sum }\underset{K}{\sum }EF_{i,j,k}\times M_{j,k}\times \;100$$

where $N_{WTT_{I}}$ denotes pollutant *i* emissions during combustion (g/km), $EF_{i,j,k}$ is the pollutant *i* emission factor (kg/kJ), and $M_{j,k}$ is fuel consumption (kJ/km). $M_{j,k}$ was calculated using the following equation:

(6) $$M_{j,k}\;=\;M\;\times \;P_{j}\;\times \;T_{j}$$

where *M* is fuel consumption (kJ/km), *P* is the proportion of fuel used, *T* is the proportion of control technology, *i* is the pollutant species, *j* is the fuel type, and *k* is the type of control technology.

Power plant, boiler combustion emissions, and emissions during transportation are the main sources of pollutant emissions in the well-to-tank phase for electric vehicles. The remainder of this section is focused on emissions caused by combustion in power plants and boilers.

Emission factor for power plants: The power plant emission factor was calculated using the following equation

(7) $$EF_{I}=EF_{I,NC}\times \;\underset{j}{\sum }\left[W_{i,j}\times \;\left(1\;-\sigma_{i,j}\right)\right]$$

where *EF_I_* is the emission factor (g/kWh), *EF_I,NC_* is the emission factor in an uncontrolled state (g/kWh), *W_i,j_* is the control technology application ratio (%), *σ_i,j_* is the pollutant removal efficiency (%), *i* is the pollutant species, and *j* is the control technology category.

Relatively small amounts of CO, VOCs, CH_4_, and N_2_O are emitted by thermal power plants in China. Therefore, measures to control these pollutants were not considered. The emission factors used were the default values from the GREET (Greenhouse gases, Regulated Emissions, and Energy use in Transportation) model. Large amounts of SO_2_, NO_x_, PM_2.5_, and PM_10_ are emitted by thermal power plants in China, and the parameters required for calculating actual emission factors for these pollutants were taken from previous publications [26,60,61,62,63].

Industrial boiler emission factors: emission factors for coal-fired industrial boilers were calculated using the method described above. Relatively small amounts CO, VOCs, CH_4_, and N_2_O are emitted by coal-fired industrial boilers; thus, measures to control these pollutants were not considered. The VOCs, CH_4_, and N_2_O emission factors were taken from previous publications [53,64]. The default CO emission factor in the GREET model was used. The SO_2_, NO_x_, PM_2.5_, and PM_10_ emission factors for boilers under an uncontrolled state with efficient removal and with control technology used were taken from previous publications [65,66,67].

Upstream emissions for individual PC and BUS in the YRD estimated using the method described above are shown in Table S9.

Elimination of motorcycles (EMC) scenario: In the YRD, MC is a major contributor of vehicular emissions. The number of MC eliminated was determined from the urban and suburban population proportions for each city [46]. In the EMC scenario, MC is assumed to be completely banned in all urban centers in the YRD by 2020. The other vehicle populations, emission factors, and vehicular emission standard implementation times were the same as those for the BAU scenario.

Integrated scenario: The integrated scenario was divided into a conservative integrated scenario (CIS) and a radical integrated scenario (RIS). The CIS combined all the control measures, and the effects of the emission reduction measures were calculated from the effects of each individual control measure. In the RIS, power for electric vehicles was assumed to be supplied by “clean” energy sources. The other parameters were the same as those for the CIS.

## 3. Results and Discussion

### 3.1. Vehicular Emission Inter-Annual Trends for Different Pollutants

**Figure 3:**
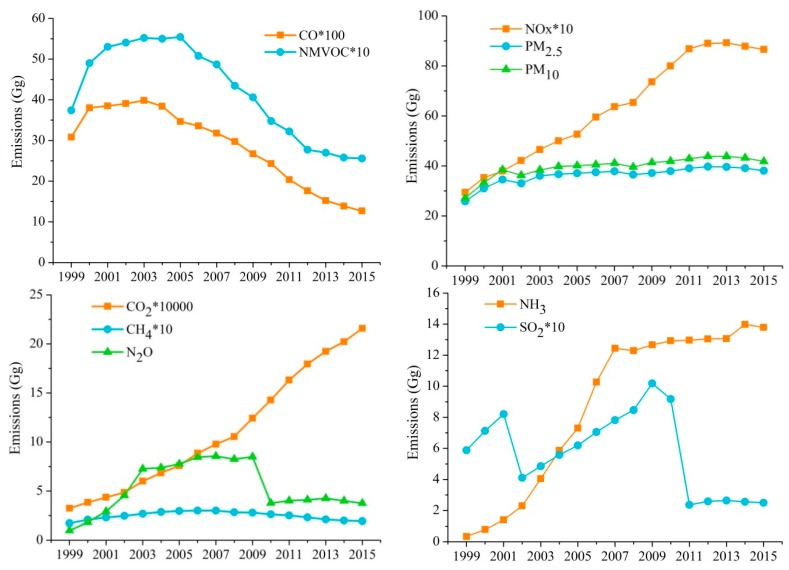
Vehicular emissions in the YRD between 1999 and 2015.

Based on the methods described above, the vehicular emissions of CO, NMVOCs, NO_x_, PM_2.5_, PM_10_, CO_2_, CH_4_, N_2_O, NH_3_, and SO_2_, widely varying in the YRD during 1999–2015, were estimated, and the inter-annual trends are shown in Figure 3.

The general trend of vehicular emissions for CO and NMVOCs increased each year by (on average) 7.04% and 8.4%, respectively, between 1999 and 2003 in the YRD. Increasingly stringent emission standards and eliminating old vehicles were then implemented, and these two pollutants’ emissions decreased from 3986.54 Gg and 554.41 Gg, respectively, in 2003 to 1268.22 Gg and 255.6 Gg, respectively, in 2015. The NO_x_, PM_2.5_, and PM_10_ emissions increased between 1999 and 2012. The NO_x_, PM_2.5_, and PM_10_ emissions in 2012 were 889.96 Gg, 39.72 Gg, and 43.82 Gg, respectively, and these were 203%, 54%, and 61% higher, respectively, than those in 1999. However, the emissions of these three vehicular pollutants decreased from 2013 to 2015 (the emissions were 865.60 Gg, 38.10 Gg, and 41.78 Gg, respectively, in 2015), which was related to the implementation of the new emissions standard, higher fuel standards, and elimination of yellow-label vehicles. Different years that began to show a decline in CO, NMVOCs, NO_x_, PM_2.5_, and PM_10_ emissions in the YRD were attributed to different major contributors of these pollutants. Passenger cars and MC were the major contributors to CO and NMVOC emissions [68], and the downtrend emissions of CO and NMVOCs were related to the rapid decrease in the MC population and stringent emission standard of PC from 2004. However, BUS and HDT were the major contributors to NO_x_, PM_2.5_, and PM_10_ emissions, and the emission downtrend of these three pollutants in 2013 may be due to the BUS and HDT in some cities (such as Shanghai, Nanjing, and Hangzhou) implementing the State IV standard from 2013.

The growth trend in vehicular CO_2_ emissions was obvious, increasing from 32349.75 Gg in 1999 to 215787.4 Gg in 2015, an increase of 567%. This increase indicates that fuel standards implemented in China were not effective in decreasing vehicular CO_2_ emissions. Furthermore, the number of PC increased markedly in China as industrialization and urbanization progressed and living standards improved. Passenger cars were the main contributors to vehicular CO_2_ emissions, and the large increases in the numbers of PC offset decreases in CO_2_ emissions by individual PC caused by the implementation of fuel standards. China has implemented new fuel standards and other measures to decrease pollutant emissions, but the limitation of the increase in PC numbers cannot be ruled out in the future. Vehicular CH_4_ emissions increased and then decreased. Vehicular CH_4_ emissions showed an upward trend from 1999 to 2007 (increased from 17.19 Gg to 30.03 Gg with an annual increase of 7.4%) and a downtrend trend between 2008 and 2015 that was related to the implementation of new emissions standards, etc. Vehicular N_2_O emissions showed a tendency to increase during the study period (from 0.979 Gg to 3.750 Gg) with an increase of 283%. However, N_2_O emissions decreased from 8.485 Gg in 2009 to 3.763 Gg in 2010. This was due to the new fuel standards which caused the fuel quality to improve. The implementation of the new fuel standards could reduce N_2_O emissions [27]. The sulfur content of gasoline decreased from 500 mg/L in 2009 to 50 mg/L in 2010 in Shanghai, and from 500 mg/L in 2009 to 150 mg/L in 2010 in Jiangsu, Zhejiang, and Anhui (Table S6). Vehicular N_2_O emissions decreased in some years but mainly followed an upward trend, because increases in the PC numbers offset decreases in emissions per PC caused by the implementation of fuel standards.

Vehicular NH_3_ emissions were found to have increased by more than the emissions of other pollutants. The NH_3_ emissions increased from 0.34 Gg to 13.79 Gg between 1999 and 2015 with an increase of 4004%. Hazy weather in China is mainly caused by PM_2.5_ [69]. The NH_3_ emitted by vehicles is essential to the formation and growth of PM_2.5_. The NH_3_ is an alkaline gas that can react with water and acidic substances (e.g., SO_2_ and NO_x_) to form major contributors to fine particles (e.g., ammonium sulfate and ammonium nitrate) [70]. Vehicle populations and traffic flows are much higher in city centers than in surrounding areas, and NH_3_ promotes hazy weather; therefore, haze is a more serious problem in city centers than in suburbs. The Chinese government and, indeed, society in general has focused very little on attention NH_3_ pollution, and vehicular NH_3_ emissions must be decreased. Vehicular SO_2_ emissions are primarily controlled by the vehicle population and sulfur content in fuel [71]. The SO_2_ emissions in the YRD changed in different ways from other pollutants between 1999 and 2015 (Figure 3). This was mainly caused by the specification of different sulfur contents in fuel standards implemented between 1999 and 2015 (Table S6). Vehicular SO_2_ emissions increased between 1999 and 2015, except for those between 2001 and 2002 and between 2010 and 2011 when SO_2_ emissions markedly decreased (from 82.02 Gg in 2001 to 41.05 Gg in 2002 and from 91.78 Gg in 2010 to 23.62 Gg in 2011, decreases of 49.9% and 74.2%, respectively).

### 3.2. Reduction Effects of Different Control Scenarios

Inventories for vehicular CO, NMVOCs, NO_x_, PM_2.5_, PM_10_, CO_2_, CH_4_, N_2_O, NH_3_, and SO_2_ emissions in the YRD in 2020 under the different emission reduction scenarios described above were calculated and compared with the vehicular emissions under the BAU scenario. The predicted vehicle population data, emission factors, and annual driving speeds for the different vehicle types will produce CO, NMVOCs, NO_x_, PM_2.5_, PM_10_, CO_2_, CH_4_, N_2_O, NH_3_, and SO_2_ emissions of 1115.8 Gg, 289.6 Gg, 840.5 Gg, 37.5 Gg, 46.4 Gg, 300785.6 Gg, 18.1 Gg, 4.4 Gg, 11.4 Gg, and 32.2 Gg, respectively, in the YRD under the BAU scenario in 2020.

**Figure 4:**
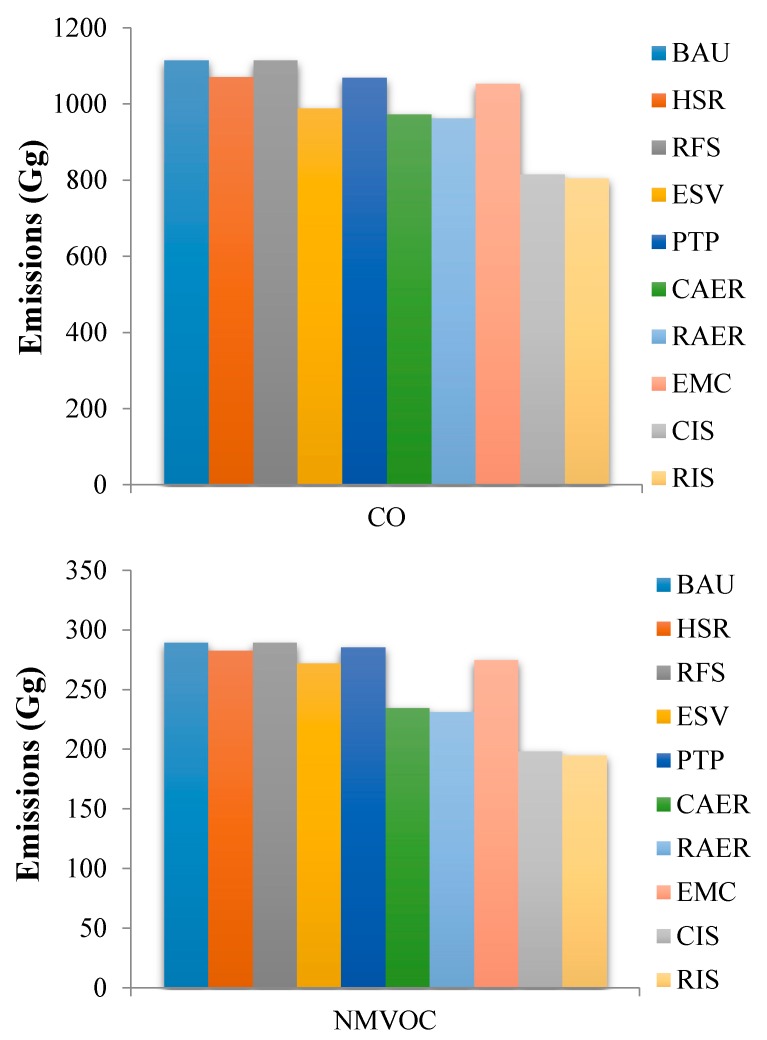
The CO and non-methane volatile organic compounds (NMVOC) emissions in 2020 under the different emission reduction scenarios.

Emissions of CO and NMVOCs will be lower in 2020 under each of the nine scenarios than under the BAU scenario (Figure 4). The CAER scenario and RAER scenario result in a greater decrease in the emission reduction effect than the other single scenarios. The CO emissions will decrease by 12.69% and 13.66% and NMVOC emissions by 18.85% and 20.03% under the CAER and RAER scenarios, respectively, relative to the BAU scenario. The CAER scenario and RAER scenario differed little in terms of decreasing emissions, indicating that CO and NMVOCs are predominantly emitted when vehicles are driven, meaning emissions are barely affected by upstream changes in power sources. The CIS scenario and RIS scenario (combining all the single reduction control scenarios together) yielded notable decreases in emissions. The CIS scenario decreased CO and NMVOC emissions by 26.91% and 31.49%, respectively, and the RIS scenario decreased CO and NMVOC emissions by 27.84% and 32.61%, respectively.

**Figure 5:**
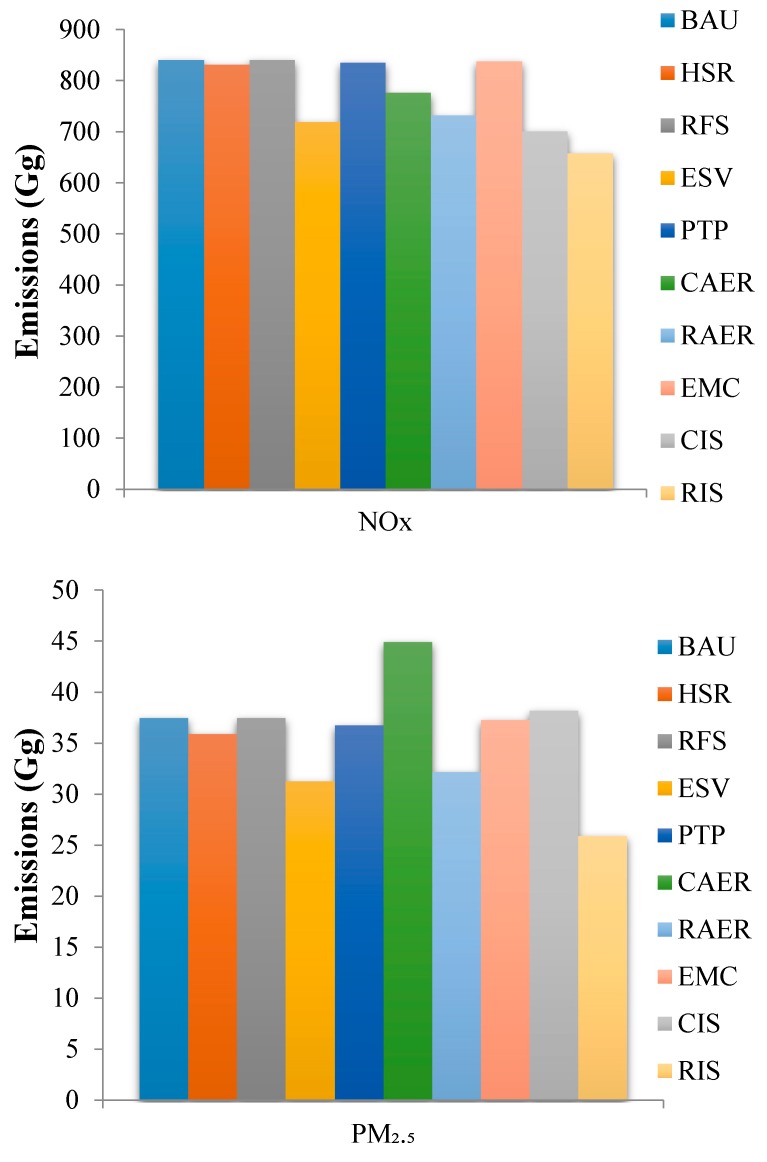

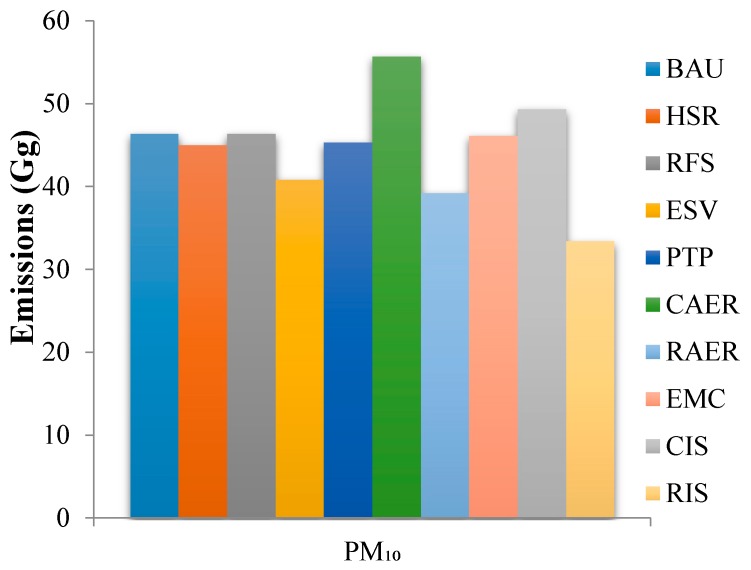
The NO_x_, PM_2.5_, and PM_10_ emissions under different emission reduction scenarios.

All nine scenarios decreased NO_x_ emissions in the YRD in 2020 relative to the BAU scenario (Figure 5). The ESV scenario decreased NO_x_ emissions by 14.41% relative to the BAU scenario, and the other single scenarios decreased NO_x_ emissions to lesser degrees. Vehicles using new energy sources (e.g., electric, hybrid, and natural gas-powered vehicles) emit less NO_x_ than traditional vehicles, and the CAER scenario and RAER scenario decreased NO_x_ emissions by 7.65% and 12.91%, respectively, relative to the BAU scenario. The emission reduction effects of the CIS scenario and RIS scenario were ideal and resulted in decreases in NO_x_ emissions of 16.57% and 21.63%, respectively, relative to the BAU scenario. Thus, the ESV scenario can cause relatively better performance for NO_x_ reduction. Additionally, the emission reduction effect of the RAER scenario was better than the CAER scenario, because the NO_x_ emissions were mainly from the upstream process and were greatly affected by upstream power composition. Thus, the synchronized promotion of upstream clean electricity can effectively promote the NO_x_ reduction effect caused by the large-scale development of electric vehicles.

All single scenarios except the CAER scenario decreased PM_2.5_ emissions in the YRD in 2020 relative to the BAU scenario (Figure 5). The PM_2.5_ emissions under the CAER scenario was 19.81% higher than that under the BAU scenario in 2020, because PM_2.5_ is predominantly emitted in the upstream phase. Coal-fired power plants are the main sources of electricity in China. Promoting electric vehicles under the CAER scenario also increased PM_2.5_ emissions. The promotion of vehicles powered by natural gas could effectively decrease PM_2.5_ emissions, but the decrease in emissions would be offset by increased upstream emissions to supply power to the electric vehicles. The proportion of “clean” energy therefore needs to increase in order to provide electricity for electric vehicles without increasing PM_2.5_ emissions. The nine scenarios had similar effects on PM_10_ emissions as the effects on PM_2.5_ emissions. The PM_10_ emissions increased by 20.07% under the CAER scenario relative to that under the BAU scenario in 2020. Promoting natural gas-powered vehicles could effectively decrease PM_10_ emissions, but the decrease in emissions would offset increased emissions upstream to supply power to electric vehicles. The RAER scenario decreased emissions from upstream power sources using “clean” energy sources, and this scenario decreased PM_10_ emissions by 15.48% compared with those under the BAU scenario.

**Figure 6:**
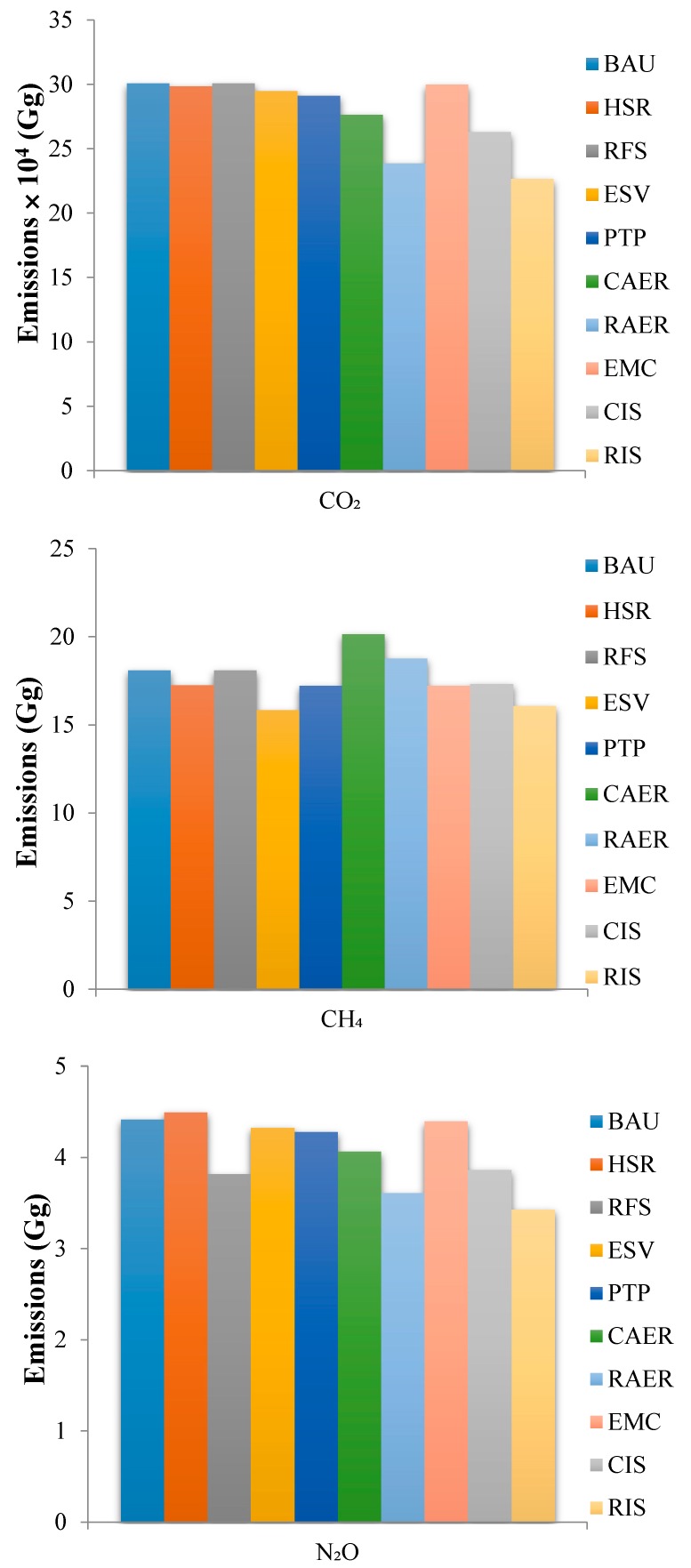
The CO_2_, CH_4_, and N_2_O emissions under different emission reduction scenarios.

The nine emission reduction scenarios decreased CO_2_ emissions relative to the BAU scenario (Figure 6). The CAER scenario and RAER scenario decreased CO_2_ emissions by 7.9% and 20.56%, respectively, relative to the BAU scenario. The large difference in the reduction effect among these two scenarios was due to the high CO_2_ being emitted upstream of electric vehicles offsetting the decreased emissions using hybrid and natural gas-powered vehicles. However, when a higher proportion of clean energy was adopted in the upstream stage, the CO_2_ emission reduction trend of electric vehicles was significant. Therefore, the emission reduction effect of the RAER scenario was relatively ideal. The CIS scenario and RIS scenario produced decreases in CO_2_ emissions of 12.53% and 24.61%, respectively, relative to the BAU scenario. The ESV scenario had the best emission reduction effect on CH_4_, decreasing by 12.49% relative to that of the BAU scenario, and the other scenarios decreased CH_4_ emissions to lesser degrees. The CAER scenario and RAER scenario increased CH_4_ emissions by 11.38% and 3.71%, respectively, relative to the BAU scenario, a finding that was related to the promotion of natural gas-powered vehicles. This means that the promotion of natural gas-powered vehicles cannot cause CH_4_ emission reduction, and the promotion of natural gas-powered vehicles in the future needs to comprehensively consider the advantages and disadvantages of pollutant emission. The CIS scenario and RIS scenario produced CH_4_ emission decreases of 4.18% and 11.19%, respectively, relative to the BAU scenario. The RFS scenario decreased N_2_O emissions by 13.61% relative to the BAU scenario. This was due to the implementation of new fuel standards, which caused the fuel quality to improve (the sulfur content of fuel strongly affects vehicular N_2_O emission). Decreasing the sulfur content of fuel is an important way to decrease vehicular N_2_O emissions [27]. The RAER scenario had the best emission reduction effect compared with the other single scenarios. However, the N_2_O emission factors for HDT and BUS increased between the State IV and State VI regulations [45], resulting in the HSR scenario increasing N_2_O emissions by 1.74% relative to the BAU scenario.

**Figure 7:**
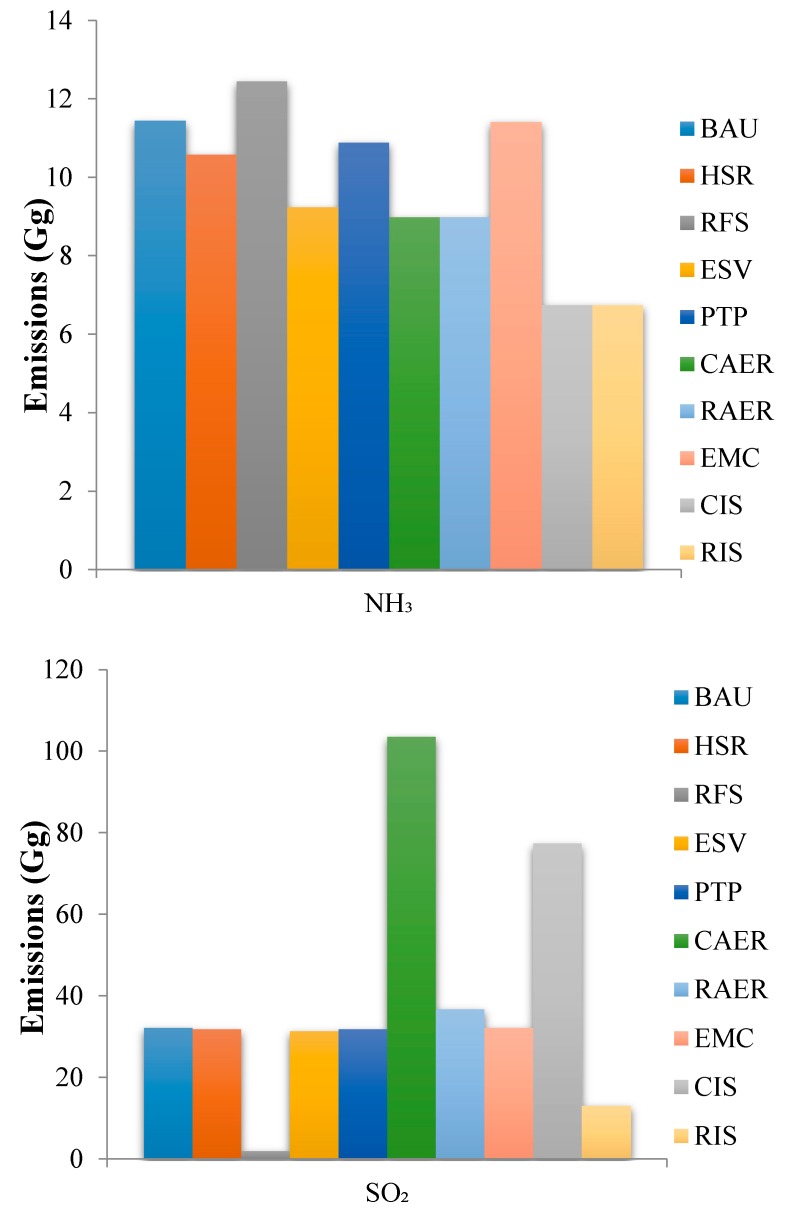
The NH_3_ and SO_2_ emissions under different emission reduction scenarios.

The different scenarios produced different reduction trends in NH_3_ emission relative to the BAU scenario except for the RFS scenario (Figure 7). The RFS scenario increased NH_3_ emissions by 8.82% relative to the BAU scenario. This was because vehicles usually use three-way catalytic converters to reduce NO_x_ to nitrogen to decrease NO_x_ emissions. However, in fact, this process very easily reduces NO_x_ to NH_3_. Three-way catalytic converters do not produce large amounts of NH_3_ when the oil quality is relatively poor, but NH_3_ emissions increase as the oil quality improves [67]. The main component of fog and haze in China is PM_2.5_ [69], and NH_3_ emitted by vehicles is essential to the formation and growth of PM_2.5_. There is a clear need to decrease NH_3_ emissions. The RFS scenario decreased SO_2_ emissions by 93.64% relative to the BAU scenario. This was a larger decrease than that for the other single scenarios because vehicular SO_2_ emissions were mainly controlled by vehicle population and the sulfur content in fuel [71]. However, the CAER scenario increased SO_2_ emissions by 221.51% relative to the BAU scenario because the upstream power for electric vehicles is mainly produced by coal-fired power plants. Promoting natural gas-powered vehicles could decrease SO_2_ emissions, but this would be offset by upstream SO_2_ emissions to produce power for electric vehicles. Thus, the RAER scenario (using “clean” energy to power electric vehicles) was much better than the CAER scenario.

### 3.3. Uncertainty Evaluation

Monte Carlo simulations were performed to estimate the uncertainties in the vehicular emissions estimated for the YRD. The distribution and coefficient of variation (CV; the standard deviation divided by the mean) of each variable were used in the Monte Carlo simulations. The variables used to calculate vehicular emissions included the vehicle population, emission factors, and VKT. The vehicle population data were obtained from official statistical yearbooks and statistical bulletins, so the uncertainty was small, and the data were assumed to have a normal distribution with a CV of 5%. The VKT data were obtained from previous publications. Kioutsioukis et al. (2004) [72] analyzed the uncertainty in global road traffic emissions and concluded that the VKT data had a normal distribution with a CV of 10%. The VKT values calculated using different methods diverged by <10% in a previous vehicular pollutant emission inventory for Shanghai [73]. Uncertainty in VKT should therefore be higher than that in the vehicle population. We assumed that the VKT data had a normal distribution with a CV of 10%. In a previous study [28], the CO, NMVOCs, and NO_x_ emission factors were found to have log-normal distributions with a CV of 17%, and the PM_2.5_ and PM_10_ emission factors were found to have log-normal distributions with a CV of 34%. There are few studies providing uncertainties in greenhouse gas (CO_2_, CH_4_, and N_2_O) and harmful gas (NH_3_ and SO_2_) emission data; therefore, we assumed that the emission factors for these species had log-normal distributions with a CV of 34%. The trial number is an important parameter that can directly affect the results of a Monte Carlo simulation. A trial number of 100,000 was used to ensure that the results were accurate.

The simulation results indicated that the ranges of uncertainty using a confidence coefficient of 95% were −14% to 19%; −23% to 31%; −21% to 27%; −29% to 43%; −29% to 43%; −30% to 49%; −29% to 47%; −33% to 58%; −44% to 80%; and −28% to 39% for CO, NMOVCs, NO_x_, PM_2.5_, PM_10_, CO_2_, CH_4_, N_2_O, NH_3_, and SO_2_, respectively. The main sources of uncertainty were variability in the data (particularly the vehicular population, VKT, and emission factors) and uncertainty in the model. Although there were uncertainties in the results, this attempt to assess the potential reduction in pollutant emissions caused by various pollutant control strategies will be useful for developing air pollution abatement strategies for the YRD.

## 4. Conclusions

In this work, ten pollutant emission inventories (i.e., CO, NMVOCs, NO_x_, PM_2.5_, PM_10_, CO_2_, CH_4_, N_2_O, NH_3_, and SO_2_) from vehicles were estimated based on the COPERT model in the YRD during 1999–2015. The vehicular emission inter-annual trends for different pollutants were analyzed based on the emission inventories. Next, the vehicular emissions of 10 pollutants were calculated under eight scenarios and the reduction effects of different control measures in 2020 were compared. Additionally, the uncertainties of the vehicular inventories were analyzed by the Monte Carlo simulation.

Influenced by the different major contributors, fuel standards, and vehicular emission standards, the pollutants (CO, NMVOC, NO_x_, PM_2.5_, PM_10_, CO_2_, CH_4_, N_2_O, NH_3_, and SO_2_) showed different time variation trends. Overall, CO_2_, which maintained a growth trend, was the major pollutant emitted from vehicles. However, NH_3_ emissions showed the most significant growth trend during the study period, and more attention should be given to this pollutant.

Relative to the BAU scenario, the ESV scenario decreased NO_x_, PM_2.5_, and CH_4_ emissions by 14.41%, 16.64%, and 12.48%, respectively; the RAER scenario decreased CO, NMVOCs, PM_10_, CO_2_, N_2_O, and NH_3_ emissions by 13.66%, 20.03%, 15.49%, 20.56%, 19.25%, and 21.46%, respectively; the RFS scenario decreased SO_2_ emissions by 93.64%, because SO_2_ emissions are primarily controlled by the sulfur content in fuel; the RIS scenario (combining all the single reduction control scenarios together) decreased CO, NMVOCs, NO_x_, PM_2.5_, PM_10_, CO_2_, N_2_O, and NH_3_ emissions more than the other scenarios.

## Supplementary Materials

The following are available online at https://www.mdpi.com/1660-4601/16/23/4790/s1, Table S1: Vehicular emission standards implementation timetable in the Yangtze River Delta, China, Table S2: The survival rates of vehicles, Table S3: Annual average vehicle kilometers travelled data for each vehicle category in the YRD (km), Table S4: The predicted value of vehicle kilometers travelled (km/year), Table S5: Vehicle categories in China corresponding with those in COPERT IV, Table S6: The sulfur content limit in gasoline and diesel in the Yangtze River Delta (mg/kg), Table S7: Vehicular emission standards implementation timetable, Table S8: The emission factors of hybrid and natural gas vehicles, Table S9: The single vehicle emissions in well-to-tank phase in 2020 (g/km).

## Nomenclature

NMVOC	non-methane volatile organic compounds
GHG	greenhouse gases
PM	particulate matter
YRD	Yangtze River Delta
GDP	gross domestic product
PC	passenger car
BUS	bus
LDV	light-duty vehicle
HDT	heavy-duty truck
MC	motorcycle
COPERT	Computer Programme to Calculate Emissions from Road Transport
BAU	business as usual
HSR	high standard replacement
RFS	raising fuel standards
ESV	elimination of substandard vehicles
PTP	public transport priority
AER	alternative energy replacement
CAER	conservative alternative energy replacement
RAER	radical alternative energy replacement
EMC	elimination of motorcycles
IS	integrated scenario
CIS	conservative integrated scenario
RIS	radical integrated scenario
VKT	vehicle kilometers travelled
GREET	Greenhouse Gases, Regulated Emissions, and Energy Use in Transportation
WTT	well-to-tank
TTW	tank-to-wheels
