# Supplementary material for: Vehicular Emission Inventory and Reduction Scenario Analysis in the Yangtze River Delta, China
Source: Int J Environ Res Public Health. 2019 Nov 29;16(23):4790. doi: 10.3390/ijerph16234790 (PMC6926843; doi:10.3390/ijerph16234790)
Supplement: Supplementary file 1 [file ijerph-16-04790-s001.pdf]

Supplementary Information

Authors:

Xiaowei Song [a,b], Yongpei Hao[a,c*]

Manuscript title:

Vehicular emission inventory and reduction scenario analysis in the Yangtze River Delta, China

Table S1: Vehicular emission standards implementation timetable in the Yangtze River Delta, China

Table S2: The survival rates of vehicles

Table S3: Annual average vehicle kilometers travelled data for each vehicle category in the YRD (km)

Table S4: The predicted value of vehicle kilometers travelled (km/year)

Table S5: Vehicle categories in China corresponding with those in COPERT Ⅳ

Table S6: The sulfur content limit in gasoline and diesel in the Yangtze River Delta (mg/kg)

Table S7: Vehicular emission standards implementation timetable

Table S8: The emission factors of hybrid and natural gas vehicles

Table S9: The single vehicle emissions in well-to-tank phase in 2020 (g/km)

Table S1. Vehicular emission standards implementation timetable in the Yangtze River Delta, China.

Regions	Vehicle types	State I	State II	State III	State IV	State V
Shanghai	PC, LDV	1999	2003	2008	2010	2014
	BUS, HDT	2001	2005	2008	2013	/
	MC	2002	2004	2009	/	/
Nanjing	PC, LDV	2000/2001	2004/2006	2008	2011	/
	BUS, HDT	2001	2005	2008	2013	/
	MC	2002	2004	2010	/	/
Hangzhou	PC, LDV	2000/2001	2004/2006	2008	2011	/
	BUS, HDT	2001	2005	2008	2013	/
	MC	2002	2004	2010	/	/
Other cities	PC, LDV	2000/2001	2004/2006	2008	2012	/
	BUS, HDT	2001	2005	2008	2015	/
	MC	2002	2004	2010	/	/

PC: passenger car, LDV: light-duty vehicle, BUS: bus, HDT: heavy-duty truck, MC: motorcycle.

Table S2. The survival rates of vehicles.

Vehicle age	PC	LDV	HDT	BUS	MC	Vehicle age	PC	LDV	HDT	BUS	MC
1	1.00	1.00	1.00	1.00	1.00	11	0.80	0.01	0.65	0.45	0.00
2	1.00	1.00	1.00	1.00	0.90	12	0.72	0.00	0.50	0.28	0.00
3	1.00	0.99	1.00	0.99	0.80	13	0.61	0.00	0.34	0.15	0.00
4	0.99	0.99	0.99	0.99	0.70	14	0.47	0.00	0.17	0.07	0.00
5	0.98	0.93	0.98	0.98	0.60	15	0.33	0.00	0.07	0.03	0.00
6	0.98	0.79	0.98	0.95	0.50	16	0.20	0.00	0.03	0.02	0.00
7	0.97	0.58	0.95	0.92	0.40	17	0.10	0.00	0.01	0.00	0.00
8	0.95	0.32	0.92	0.85	0.30	18	0.04	0.00	0.01	0.00	0.00
9	0.92	0.10	0.85	0.75	0.20	19	0.02	0.00	0.00	0.00	0.00
10	0.87	0.03	0.78	0.61	0.10	20	0.00	0.00	0.00	0.00	0.00

Table S3. Annual average VKT vehicle kilometers travelled data for each vehicle category in the YRD (km).

City	Fleet	1999	2007	2015
Shanghai	PC	30900	22581	18500
	LDV	21000	39000	37000
	BUS	35000	57000	62000
	HDT	20200	21671	19200
	MC	8554	8959	5200
Nanjing	PC	36025	27000	21500
	LDV	22000	34000	42000
	BUS	38000	57000	70000
	HDT	40000	52000	66000
	MC	15000	11000	5500
Hangzhou	PC	36025	27000	21500
	LDV	28000	37500	46000
	BUS	38000	57000	70000
	HDT	33000	43000	55500
	MC	14000	10000	4500
Hefei	PC	29000	25000	18000
	LDV	22000	36000	40500
	BUS	30000	45000	65000
	HDT	32000	52000	56000
	MC	13000	9500	3000

Table S4. The predicted value of VKT vehicle kilometers travelled (km/year).

City	Type	2016	2017	2018	2019	2020
Shanghai	PC	18000	17500	17000	16500	16000
	LDV	37500	38000	38500	39000	39500
	BUS	62500	63000	63500	64000	64500
	HDT	19500	19700	19900	20100	20300
	MC	5000	4800	4500	4300	4000
Nanjing[a]	PC	20000	19500	19000	18500	18000
	LDV	43000	44000	45000	46000	47000
	BUS	70600	70700	70800	70900	71000
	HDT	67000	67500	68000	68500	69000

	MC	5300	5200	5100	5000	4900
Hangzhou[b]	PC	20000	19500	19000	18500	18000
	LDV	47000	48000	49000	50000	51000
	BUS	70600	70700	70800	70900	71000
	HDT	56000	56500	57000	57500	58000
	MC	4400	4300	4200	4100	4000
Hefei[c]	PC	17500	17300	17100	16900	16700
	LDV	41000	41500	42000	42500	43000
	BUS	67800	68100	68400	68700	69000
	HDT	57000	58000	59000	60000	61000
	MC	3700	3600	3500	3400	3300

a Nanjing, Wuxi, Changzhou, Suzhou, Nantong, Yancheng, Yangzhou, Zhenjiang, Tai'zhou
b Hangzhou, Ningbo, Jiaxing, Huzhou, Shaoxing, Jinhua, Zhoushan, Taizhou
c Hefei, Wuhu, Maanshan, Tongling, Anqing, Chuzhou, Chizhou, Xuancheng

Table S5. Vehicle categories in China corresponding with those in COPERT Ⅳ.

**Categories of Chinese vehicles**	**Vehicle categories by COPERT Ⅳ**
Big-size passenger cars<br>Middle-size passenger cars	Big-size passenger cars
Small-size passenger cars<br>Mini passenger cars	Small-size passenger cars
Heavy-duty vans<br>Intermediate duty vans	Heavy-duty vans
Light-duty vans<br>Mini vans	Light-duty vans
Motorcycles	Motorcycles

Table S6. The sulfur content limit in gasoline and diesel in the Yangtze River Delta (mg/kg).

Fuel	Region	1999	2000	2001	2002	2003	2004	2005	2006	2007	2008	2009	2010	2011	2012	2013	2014	2015
Gasoline	shanghai	1500	1000	800	800	800	800	800	500	500	500	500	50	50	50	50	10	10
	Other	1500	1000	1000	1000	800	800	800	500	500	500	500	150	150	150	150	50	50
Diesel	Shanghai	5000	5000	5000	2000	2000	2000	2000	2000	2000	2000	2000	50	50	50	50	10	10
	Other	5000	5000	5000	2000	2000	2000	2000	2000	2000	2000	2000	2000	350	350	350	350	50

Table S7. Vehicular emission standards implementation timetable.

City	Vehicle types	State Ⅴ	State Ⅵ
Shanghai	PC, LDV	2014	2019
	BUS, HDT	20170101<br>20170701	/
Nanjing[a]	PC, LDV	20160401<br>20180101	2020
	BUS, HDT	20170101<br>20170701	/
Hangzhou[b]	PC, LDV	20160401<br>20180101	2020
	BUS, HDT	20170101<br>20170701	/
Hefei[c]	PC, LDV	20170101<br>20180101	2020
	BUS, HDT	20170101<br>20170701	/

a Nanjing, Wuxi, Changzhou, Suzhou, Nantong, Yancheng, Yangzhou, Zhenjiang, Tai'zhou
b Hangzhou, Ningbo, Jiaxing, Huzhou, Shaoxing, Jinhua, Zhoushan, Taizhou
c Hefei, Wuhu, Maanshan, Tongling, Anqing, Chuzhou, Chizhou, Xuancheng

Table S8. The emission factors of hybrid and natural gas vehicles.

Vehicle type	Fuel type	Emission factor (g·km^{-1})				
		CO	NMVOC	NO_x	$PM_{2.5}$	PM_{10}
PC	hybrid	0.3517	0.1174	0.0193	0.0156	0.0189
	natural gas	0.8858	0.0435	0.1494	0.009	0.0255
BUS	hybrid	1.1627	0.4700	10.6600	0.1400	0.1900
	natural gas	1.8953	0.0399	5.8500	0.0480	0.0670
**Vehicle type**	**Fuel type**	**Emission factor (g·km^{-1})**				
		CO_2	CH_4	N_2O	NH_3	SO_2
PC	hybrid	107.4000	0.0000	0.0030	0.0010	0.1240
	natural gas	113.6100	0.0093	0.0000	0.0000	0.0000
BUS	hybrid	786.2700	0.0000	0.0300	0.0100	0.2150
	natural gas	955.0000	1.1500	0.0000	0.0000	0.0000

Table S9. The single vehicle emissions in well-to-tank phase in 2020 (g/km).

Vehicle type	CO	NMVOC	NO_x	$PM_{2.5}$	PM_{10}	CO_2	CH_4	N_2O	SO_2
PC	0.0313	0.0153	0.182	0.0432	0.0585	171.77	0.006	0.006	0.2837
BUS	0.1744	0.0780	0.8849	0.2157	0.4157	852.632	0.04	0.04	1.4122
